# Traditional chinese medicine to prevent and treat diabetic erectile dysfunction

**DOI:** 10.3389/fphar.2022.956173

**Published:** 2022-09-21

**Authors:** Yanfei Feng, Tianhao Shi, Yuli Fu, Bodong Lv

**Affiliations:** ^1^ The Second School of Clinical Medicine, Zhejiang Chinese Medical University, Hangzhou, China; ^2^ Zhejiang Province Key Laboratory of Traditional Chinese Medicine (Laboratory of Andrology), Hangzhou, China; ^3^ Department of Urology, The Second Affiliated Hospital, School of Medicine, Zhejiang University, Hangzhou, China

**Keywords:** diabetic erectile dysfunction, traditional Chinese medicine, Chinese herbs, endothelial dysfunction, vasculopathy, oxidative stress

## Abstract

Diabetic erectile dysfunction (DED) is one of the most common complications of diabetes mellitus. However, current therapeutics have no satisfactory effect on DED. In recent years, traditional Chinese medicine (TCM) has shown good effects against DED. By now, several clinical trials have been conducted to study the effect of TCM in treating DED; yet, the underlying mechanism is not fully investigated. Therefore, in this review, we briefly summarized the pathophysiological mechanism of DED and reviewed the published clinical trials on the treatment of DED by TCM. Then, the therapeutic potential of TCM and the underlying mechanisms whereby TCM exerts protective effects were summarized. We concluded that TCM is more effective than chemical drugs in treating DED by targeting multiple signaling pathways, including those involved in oxidation, apoptosis, atherosclerosis, and endothelial function. However, the major limitation in the application of TCM against DED is the lack of a large-scale, multicenter, randomized, and controlled clinical trial on the therapeutic effect, and the underlying pharmaceutical mechanisms also need further investigation. Despite these limitations, clinical trials and further experimental studies will enhance our understanding of the mechanisms modulated by TCM and promote the widespread application of TCM to treat DED.

## Introduction

Erectile dysfunction (ED) is one of the secondary complications of diabetes mellitus (DM) and affects >37% of men with DM (Maiorino et al., 2017), more than three times of the prevalence in men without DM (Malavige and Levy, 2009). According to a recent study, the estimated prevalence of DM is approximately 12.4% in China (Wang L. et al., 2021). Furthermore, the prevalence of DM between 2021 and 2045 is estimated to be 21.1% in middle-income countries, including China (Sun et al., 2022). Therefore, a large number of men in China will presumably suffer from the high medical costs and poor life quality associated with diabetic ED (DED). Although the association between DM and the development of ED has been documented since 1970s, the mechanism underlying DED has not been fully characterized yet (Gur et al., 2014). Increasing attention has been given to ED in men with DM due to the multifactorial pathophysiology involved, including neuropathy, macrovascular arterial disease, structural remodeling of the corporeal tissue, and hormonal imbalance (Kamenov, 2015).

Currently, the most commonly used method in the treatment of ED is chemical drugs therapy; however, this treatment has limitations in treating DED (Kamenov, 2015). Phosphodiesterase type 5 (PDE5) inhibitors currently represent the first-choice treatment strategy for ED. However, the beneficial effect of this treatment is lower in diabetic than in non-diabetic subjects (Bruzziches et al., 2008). It has been reported that sildenafil, a commonly used inhibitor of PDE5, can improve the sexual function in approximately 63% of DM patients, whereas beneficial effects have been observed in approximately 83% of non-diabetic subjects (Fink et al., 2002). The reduced responsiveness to PDE5 inhibitors in men with DM may be related to the severity of neuropathy (Musicki and Burnett, 2007). Additionally, PDE5 inhibitors can induce adverse effects, such as facial redness, headache, or gastrointestinal reactions (Condorelli et al., 2013; Vita et al., 2019). Therefore, a novel therapeutic approach is urgently needed.

In recent years, traditional Chinese medicine (TCM) has shown good effects against DED (Yin and Fang, 2019). According to a previous study, combined use of PDE5 inhibitors with TCM can promote the blood circulation, and the clinical efficacy obtained is often higher than that seen with the use of PDE5 inhibitors alone (Luo et al., 2019). Therefore, an in-depth understanding of the molecular mechanisms underlying DED is required to establish new treatment methods and targets that can improve the life quality of DM patients.

We searched the electronic databases of PubMed, Embase, the Cochrane Library and China National Knowledge Infrastructure (CNKI) via the following terms: 1) “Traditional Chinese medicine” or “Chinese medicine” or “herb” or “Chinese herb” and 2) “diabetic erectile dysfunction” or “diabetes erectile dysfunction” or “erectile dysfunction”. The search was limited to the studies published in English or Chinese. The final literature search was performed on May 5, 2022.

In this review, we summarize the current understanding of the molecular mechanisms underlying DED, including the risk factors and pathogenesis and discussed the therapeutic effect of TCM on DED.

### Risk of DED

Numerous risk factors, including aging, duration of DM, poor glycemic uncontrol, decreased physical activity, and pharmaceutical use, have been proposed to be involved in the development of DED (Phe and Roupret, 2012; Hatzimouratidis and Hatzichristou, 2014). A cross-sectional study has reported that the prevalence of DED is 4.6 and 45.5% for men aged 20–29 and 60–69 years, respectively (Fedele et al., 1998). Additionally, the prevalence of ED in men aged 21–30, 50–60, and >60 years has been reported to be 1.1, 55, and 75%, respectively, in another cohort of DM patients (Klein et al., 1996). Furthermore, the ED risk is higher in men with a long history of DM compared with men without DM (Bacon et al., 2002). A similar situation was observed in another cross-sectional study, in which the prevalence was also significantly higher in patients with DM for >6 years than in those with DM for ≤5 years (Fedele et al., 1998).

There is a link between glycemic control and DED. It has been reported that there is approximately 2–5-fold increased risk in ED patients without glycemic control compared with the patients whose glycemia is well controlled (Fedele et al., 2000). Although the mechanism whereby poor glycemic control induces ED in men with DM is still unclear, a recent study has reported that glycemic control can prevent ED *via* ameliorating endothelial damage and promoting vasculogenesis (Castela et al., 2017). Additionally, lack of physical activity has been shown to be associated with high prevalence of ED in men with DM (Zheng et al., 2006), and high physical activity reduces the risk of having ED (Cheng et al., 2007).

The hyperglycemia that accompanies DM is also an independent risk factor for ED among men with DM. Recent evidence has suggested that chronic hyperglycemia contributes to downregulating target-derived nerve growth factor (NGF), which is a trophic factor essential for the survival of small-diameter primary sensory neurons and autonomic nerves (Kim et al., 2009). NGF has been reported to significantly improve diabetes-induced ED through stimulation of testosterone biosynthesis. Finally, many drugs, including anti-hypertensives and psychiatric agents, which are commonly used in the treatment of DM, also contribute to the development of ED (Phe and Roupret, 2012).

### Pathogenesis of DED

**FIGURE 1:**
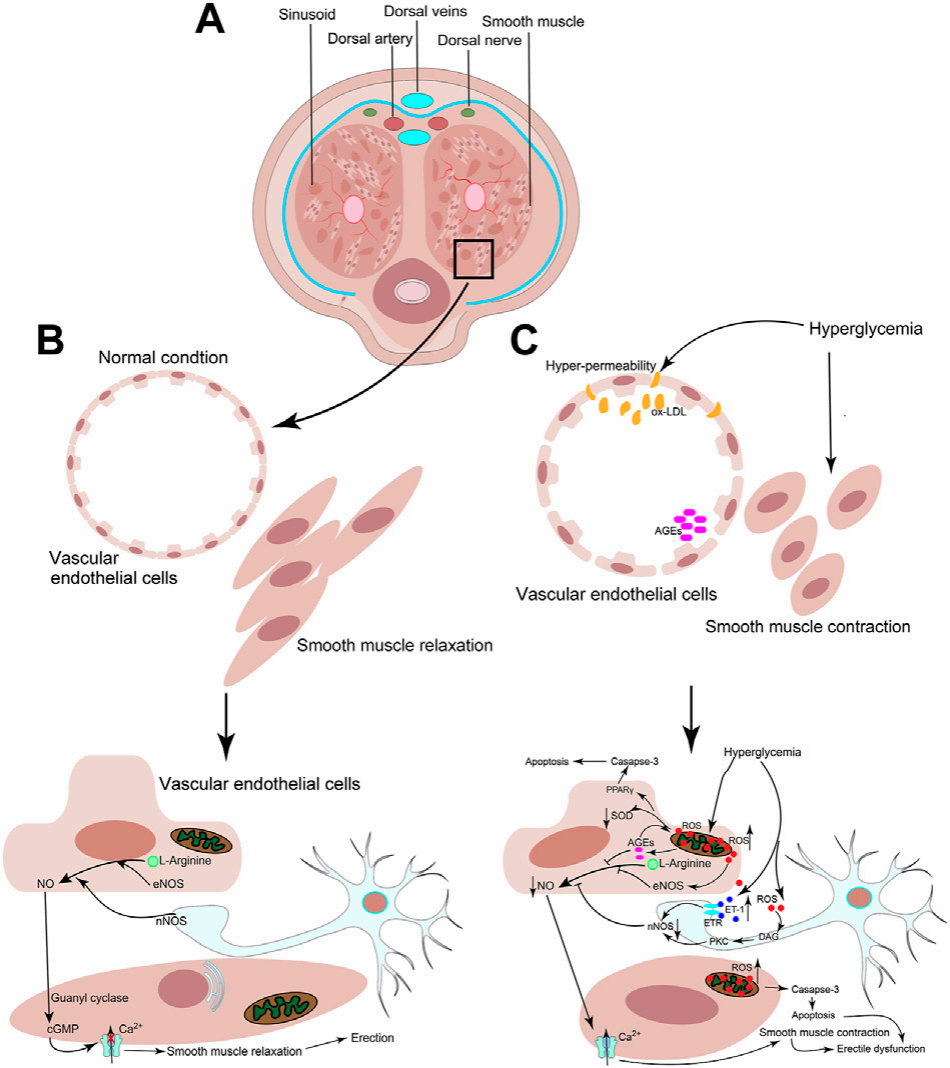
Schematic of the mechanisms involved in the etiology of diabetic vascular complications. **(A)** Cross-sectional pattern of the penis. **(B)** Under the physiological conditions, nitric oxide (NO) is generated by the corporal endothelial cells and stimulates the smooth muscle to produce cyclic guanosine monophosphate (cGMP), which dilates the vascular smooth muscle of the corpora cavernosa and thereby facilitates the blood supply resulting in erection. **(C)** Hyperglycemia promotes oxidative stress and the formation of advanced glycation end products (AGEs) and oxidized low-density lipoprotein (ox-LDL). Consequently, the permeability of the vascular endothelium and the production of reactive oxygen species (ROS) are increased. ROS can downregulate NO via inhibiting the activity of endothelial nitric oxide synthase (eNOS). Additionally, the activation of the polyol pathway impairs neurovascular function via suppressing the activity of neuronal nitric oxide synthase (nNOS). Further, the accumulation of AGEs and ox-LDL causes vascular thickening and atherosclerosis in the cavernous sinusoid. Finally, the prolonged oxidative stress can also induce apoptosis.

The development of DED is a complex and multifactorial process. The process appears to be affected by multiple physiological and psychological factors. The physiological factors include endothelial dysfunction, oxidative stress, and vascular and neurogenic causes. The psychological factors are depressive symptoms, anxiety, and decreased libido (Phe and Roupret, 2012). The multiple mechanisms involved in DED are illustrated in Figure 1.

### Physiological factors

#### Endothelial dysfunction

Endothelial dysfunction is defined as the failure of the vascular endothelium to subserve its normal role in vasodilatation and/or vascular homeostasis which is considered as an important component of DED (Hatzimouratidis and Hatzichristou, 2014). Multiple aspects of endothelial function are affected in DM, including impaired intercellular junctions of endothelial cells (ECs) (Rho et al., 2017), decreased nitric oxide (NO) bioavailability, and altered production of advanced glycation end-products in ECs (Phe and Roupret, 2012; Kamenov, 2015).

It is well known that the vascular endothelium is a self-balancing tissue of the body and regulates vascular tension and structure. However, hyperglycemia can induce a significant increase in vascular permeability via downregulating vascular endothelial-cadherin, which is an important adherent junction protein that maintains cell polarity and integrity (Li et al., 2019). In turn, hyper-permeability of the vascular endothelium in the cavernous sinusoid and cavernous artery can promote the production of oxidized low-density lipoprotein (ox-LDL) to induce ED (Ryu et al., 2013).

Another important mechanism involved in DED is mediated through the endothelium by the action of NO. It is well known that the physiological erectile function requires various cell types. Firstly, nitric oxide (NO) is generated by corporal ECs, and the erectile response is triggered by the initial release of NO from the autonomic dilator nerve fibers. Then, NO stimulates the smooth muscle to produce cyclic guanosine monophosphate (cGMP), which dilates the vascular smooth muscle of the corpora cavernosa and thereby facilitates the blood supplying into cavernous sinus of penis, resulting in erection (Kavoussi et al., 2015). It has been reported that NO production is markedly decreased in the penis of diabetic rats Escrig et al., 2002). Additionally, supplement of l-arginine, a precursor of NO, could significantly improve erectile functions in DM patients [29]. It is well known that NO is produced by endothelial NO synthase (eNOS) and neuronal NO synthase (nNOS) in the corpora cavernosa (Burnett, 2006). Studies have shown that the eNOS level and nNOS-mediated cavernosal smooth-muscle relaxation in diabetic animals are reduced (Cartledge et al., 2001b; Toque et al., 2011). Although the mechanism underlying the downregulation of eNOS and nNOS in DM is not fully known, the hyperglycemia-induced overproduction of ROS may downregulate eNOS and nNOS (Koo and Vaziri, 2003). Additionally, endothelin (ET-1), which is upregulated in patients with DM, can suppress eNOS and downregulate NO via activating the RhoA/Rho-kinase pathway (Kalani, 2008; Kamenov, 2015).

Advanced glycation end products (AGEs) have been reported to accumulate in the corpus cavernosum of DM patients (Hatzimouratidis and Hatzichristou, 2014). The accumulation of AGEs can impair the endothelial and neuronal NO-mediated relaxation of the cavernosal smooth muscle (Cartledge et al., 2001b). Furthermore, AGEs can also form covalent bonds with vascular collagen, an interaction that increases vascular permeability and endothelial expression of adhesion molecules, thereby leading to vascular pathology (Wen et al., 2002). Accordingly, inhibitors of AGE formation can reverse the ED in diabetic rats (Usta et al., 2006).

### Vasculopathy

Diabetic vasculopathy plays a role in the pathophysiology of ED. As discussed above, the process of erection needs the blood flow into the corpora cavernosae and cavernosal spaces. However, vascular structural changes have been described in men with DM (Hatzimouratidis and Hatzichristou, 2014). It has been reported that atherosclerosis and plaque formation, which are found in DM patients, can lead to ED due to penile macrovascular occlusion (Montorsi et al., 2005). Additionally, increased synthesis of the connective tissue of the smooth muscle in the corpus cavernosum can also induce peripheral vascular disease, which leads to nerve ischemia and damage (Malavige and Levy, 2009). The mechanism of diabetic vasculopathy is associated with endothelial dysfunction, since AGE accumulation results in vascular thickening and atherosclerosis (Singh et al., 2001).

### Neuropathy

Neuropathy often accompanies DM and is a significant factor to the pathophysiology of DED. It is known that the erectile process initially requires an autonomic neural input to direct the blood flow into the corpora cavernosae, and thus the autonomic nervous system is involved in this process (Dey and Shepherd, 2002). Then, the peripheral nervous system, including the pudendal nerve, is activated to induce the contraction of the bulbo-cavernous and ischiocavernosus muscles, whereby the venous outflow from the corpora cavernosa is inhibited (Defeudis et al., 2022). Therefore, the autonomic and peripheral nervous systems both participate in the erection process, and both autonomic and peripheral neuropathy occur in men with DM.

Autonomic neuropathy in DED is characterized by the reduced or absent parasympathetic activity needed. Parasympathetic activity is necessary to relax the smooth muscle of the corpus cavernosum, and the impaired parasympathetic activity in DED is associated with a decrease in NOS activity (Bleustein et al., 2002). Another study has found that patients with DM have reduced levels of norepinephrine and acetylcholinesterase in the corpus cavernosum tissue, and thus the autonomic-nerve–mediated relaxation of the penile smooth muscle is decreased (Saenz de Tejada et al., 1989). Furthermore, the peripheral neuropathy induced by DM can impair the sensory impulses from the shaft and the glans of the penis to the reflexogenic erectile center, and thus the contractile force of the bulbocavernosus and ischiocavernosus muscles is reduced (Herman et al., 2012). It has been reported that the dysfunction of the penile nerves in some men with DM was easier damaged when compared to the other peripheral nerves (Bleustein et al., 2002). According to previous studies, the pathophysiology of neuropathy involves several factors, including increased oxidative stress, accumulation of AGEs, and decreased NOS activity (Moore and Wang, 2006; Malavige and Levy, 2009).

### Oxidative stress

Oxidative stress is easily triggered under hyperglycemia, which leads to the accumulation of reactive oxygen species (ROS) since the levels of antioxidants are reduced in DM patients (Cellek et al., 1999). In addition, mitochondrial respiratory chain leakage, NOS, and elevated xanthine oxidase activity all increase ROS levels (Cameron and Cotter, 2007). In turn, ROS can affect nitrergic neurons and endothelial cells and also change NOS expression and activity through multiple signaling pathways.

Increased production of ROS can accelerate the formation of AGEs and lipid peroxidation (Osawa and Kato, 2005). In turn, AGEs can upregulate adhesion molecules that mediate vascular damage and impair the relaxation of the smooth muscle in the corpus cavernosum of diabetic rats (Cartledge et al., 2001a). Additionally, AGEs have been shown to generate ROS and quench NO, thereby impairing the function of the endothelium (Soro-Paavonen and Forbes, 2006) and nitrergic relaxations (Cellek et al., 1999). Additionally, AGEs, in turn, induce ROS generation via disrupting mitochondrial electron transport chain and increasing NADPH oxidase activity (Lin et al., 2012). Furthermore, ROS can also interact with NO to form peroxynitrite and thereby reduce the available NO concentration. In addition, peroxynitrite has been reported to play a central role in atherogenesis via producing oxidized LDL (Pacher and Szabo, 2006). Third, the increase in ROS levels, particularly in the form of hydroxyl radicals or peroxynitrite, which cause cell damage and death, has been reported to increase the incidence of endothelial apoptosis, thereby leading to the denudation of the endothelium and further reduction in available NO (Hu et al., 2016). Fourth, Oxidative stress can stimulate the accumulation of glucose metabolites, which upregulate diacylglycerol (DAG), and DAG activates protein kinase C(PKC) (Cameron and Cotter, 2007). PKC activates several mechanisms that can modulate neurovascular activity via activating mitogen-activated kinases (MAPK) (Brownlee, 2005). MAPK inhibitors have been reported to improve somatic nerve function in diabetic rats (Price et al., 2004). Finally, it has been reported that superoxide anion, which is a type of ROS, has a direct vasoconstriction effect through mobilization of calcium ions (Katusic and Vanhoutte, 1989), whereby ED may be induced.

As discussed above, oxidative stress can lead to DED *via* multiple mechanisms. Therefore, elimination of oxidative stress is a promising therapeutic approach to treat DED. In fact, there are several anti-oxidant enzymes (especially vitamin E) that are extensively used in the treatment of DED. Vitamin E presumably attenuates diabetes-induced defective relaxations (Keegan et al., 1995). Other studies have suggested that vitamin E inhibits monocyte adhesion, cytokine release, and platelet adhesion via inhibiting PKC activation (Devaraj et al., 1996; Ganz and Seftel, 2000). However, the role of vitamin E needs to be further quantified in both animal and clinical studies before it can be used in humans.

### Apoptosis

Apoptosis is also another factor underlying DED (Tarhan et al., 2009). It has been shown that the numbers of both endothelial and smooth-muscle cells are reduced and the ratio of TUNEL-positive cells in the corpus cavernosum is also increased in a mouse model of DED (Li et al., 2018). Consequently, eNOS is downregulated, and the smooth-muscle is damaged, whereby the relaxation function of the corpus cavernosum is impaired, and penile erection is diminished.

According to previous studies, multiple signaling pathways have been proposed to be involved in diabetes-induced apoptosis in the penile tissue (Zhang Z. et al., 2019). First, the mitogen-activated protein kinase (MAPK) signaling pathway is closely related to the occurrence and development of diabetes-induced ED. MAPK participates in various biological functions, including cell proliferation and apoptosis, and MAPK is activated under the condition of high glucose (Zhang Z. et al., 2019). The activation of MAPK can activate the downstream “peroxisome proliferators-activated receptor gamma” (PPARγ) to induce the apoptosis of vascular smooth muscle cells (Wan et al., 2014), and inactivation of the MAPK/PPARγ signaling alleviates diabetes-induced ED via suppression of the corpus cavernosal cell apoptosis (Zhang Z. et al., 2019).

Furthermore, oxidative stress induced by hyperglycemia triggers apoptosis. Nuclear factor E2-related factor 2 (Nrf2), which attenuates oxidative-stress–induced injury by upregulating several antioxidant genes, is downregulated in the corpus cavernosum of DED animal model, and enhanced expression of Nrf2 can ameliorate the erectile dysfunction (Wang et al., 2020). Additionally, ER stress activated by hyperglycemia can also increase the percentage of apoptotic cells in the corpus cavernosum of mice with DED, in association with decreased SOD activity (Zhang et al., 2018).

### Psychological factors

Men with DM can have more psychosexual issues and easier to develop ED compared with men without DM, since DM is associated with depression. It demonstrated that the prevalence of depressive symptoms was high in DM patients (Anderson et al., 2001). Furthermore, DED is significantly associated with a disturbed mental state (Penson et al., 2003), and thus there is a possible reinforcing mechanism of the development of DED and depressive symptoms. Furthermore, the reduction in erectile function because of physical changes associated with DM can generate anxiety, worsening condition. A study demonstrated that DED is strongly associated with premature ejaculation and reduced libido, underlining the importance of possible psychological factors (Malavige et al., 2008). This hypothesis needs to be tested in men with DM, who have many factors that can potentially increase sexual inhibition and reduce excitation.

### Short summary of the progress on deciphering DED pathophysiology

Considering the complex mechanism of DED, multiple signaling pathways and processes are involved in the pathogenesis of DED. One of the most important is that these processes do not function alone and are affected by each other. According to a previous report, oxidative stress might be the first aspect triggered under hyperglycemia (Monnier et al., 2006). Subsequently, oxidative stress can affect endothelial function via reducing NO production and accelerating AGE accumulation. Additionally, AGEs can also stimulate the generation of ROS. Therefore, oxidative stress and AGEs reciprocally activate each other under hyperglycemia. In addition, AGEs can also lead to vasculopathy via inducing vascular thickening and atherosclerosis and result in neuropathy via impairing smooth-muscle relaxation. Further, chronic oxidative stress can also induce apoptosis and fibrosis via activating multiple signaling pathways. Finally, cavernous smooth muscle and endothelial cell apoptosis further reduce NO level. However, the link among these different aspects has not yet been fully investigated. Furthermore, psychological factors, such as less frequent erection, can also impair endothelial function.

### Clinical evaluations of the effects of TCM on DED

TCM has been used for the effective treatment of DM and associated complications, with few side effects, for a long time in the Chinese history (Wang et al., 2018). There are at least three different types of TCM, including TCM formulae, crude extracts of herbs, and monomers of Chinese herbs. To date, the efficacies of several TCM formulae and crude extracts of herbs in treating DED have been assessed in randomized controlled trials.

Buyang Huanwu Decoction (BYHWD) is a classic traditional TCM formula and has 13 Chinese medicines components (Table 1). A randomized control trial showed that the International Index of Erectile Function-5 (IIEF-5) and World Health Organization Quality of Life Scale (WHOQOL-BREF) scores in the DED patients treated with BYHWD were both higher than those in the DED patients treated with vitamin B1 tablets (Yang et al., 2021). Furthermore, the concentration of testosterone was higher and the expression levels of estradiol (E2), follicle-stimulating hormone (FSH), and luteinizing hormone (LH) were lower in the DED patients treated with BYHWD.

**TABLE 1:** Summary of the prescribed formulae and natural products from Traditional Chinese medicine used in clinical trials.

Name	Dosage Form	Species, concentration, source	Research design	Grouping and number of people		Treatment method		Treatment	Quality control reported	References
				Treatment group	Control group	Treatment group	Control group	Duration		
Buyang Huanwu Decoction (BYHWD)	Decoction	*Astragalus mongholicus* Bunge (30 g), *Paeonia lactiflora* Pall. (12 g), *Angelica sinensis* (Oliv.) Diels (12 g), *Conioselinum anthriscoides* ‘Chuanxiong’ (12 g), *Carthamus tinctorius* L. (12 g), *Prunus davidiana* (Carrière) Franch. (12 g), *Pheretima aspergillum* (12 g); *Cornus officinalis* Siebold & Zucc.(15 g); *Dioscorea polystachya* Turcz. (15 g); *Bupleurum chinense* DC. (12 g); *Rehmannia glutinosa* (Gaertn.) DC. (25 g); *Pueraria montana* (Lour.) Merr. (15 g); *Panax notoginseng* (Burkill) F.H.Chen (10 g). Prepared by Yang et al., 2021	Randomized controlled experiment	33	35	BYHWD (1 prescription/day) + Vitamin B1 tablets (10 mg/day)	Vitamin B1 tablets (10 mg/day)	4 months	Prepared according to China Pharmacopoeia	Yang et al. (2021)
Huoxue Tongluo Qiwei Decoction (HXTLQWD)	Decoction	*Paeonia lactiflora* Pall. (30 g), *Cyathula officinalis* K.C.Kuan (15 g), *Scolopendra subspinipes mutilans* L. Koch (3 g), *Hirudo nipponica* Whitman. (6 g), *Angelica sinensis* (Oliv.) Diels (12 g), *Gypsophila vaccaria* (L.) Sm. (20 g), *Epimedium brevicornu* Maxim. (20 g), *Gynochthodes officinalis* (F.C.How) (20 g)*, Curcuma aromatica* Salisb. (15 g), *Citrus × aurantium* L. (10 g)*, Bupleurum chinense* DC. (12 g), *Tribulus terrestris* L (15 g). Purchased by Beijing Kangrentang Pharmaceutical Co., Ltd	Randomized controlled experiment	33	33	HXTLQWD(two bags/day)+Tadalafil (5 mg/day)	Tadalafil (5 mg/day)	4 weeks	Prepared according to China Pharmacopoeia	Sun et al. (2021)
Yishen Huoxue Decoction (YSHXD)	Decoction	*Lycium barbarum* L. (15 g), *Astragalus mongholicus* Bunge (20 g), *Rehmannia glutinosa* (Gaertn.) DC. (15 g), *Rubus idaeus* L. (10 g), *Cuscuta chinensis* Lam. (10 g), *Ligustrum lucidum* W.T.Aiton (10 g), *Allium tuberosum* Rottler ex Spreng. (10 g), *Morus alba* L. (15 g), *Rhodiola rosea* L. (15 g), *Angelica sinensis* (Oliv.) Diels (15 g), *Paeonia lactiflora* Pall. (12 g), *Dioscorea tokoro* Makino ex Miyabe (20 g), *Polygonatum sibiricum* Redouté (20 g), *Plantago asiatica* L. (10 g), *Ostrea gigas* Thunberg (30 g)*, Achyranthes bidentata* Blume (10 g). Prepared by Li et al., 2021	Randomized controlled experiment	30	30	YSHXD (1 prescription/day)	Levocarnitine oral Solution (10 ml/day)	3 months	Prepared according to China Pharmacopoeia	Li et al. (2021)
			Randomized controlled experiment	56	28	YSHXD (1 prescription/day) + Hypoglycemia agent	Hypoglycemia agent		Prepared according to China Pharmacopoeia	Li et al. (2011)
			Randomized controlled experiment	58	58	YSHXD (1 prescription/day) + Thioctic acid injection (20 ml, 0.6 g/daily)	Thioctic acid injection (20 ml, 0.6 g/day)	12 weeks	Prepared according to ChinaPharmacopoeia	Zhang and Qiu, (2017)
Yiqi Yangyin Huoxue Decoction (YQYYHXD)	Decoction	*Astragalus mongholicus* Bunge (30 g), *Codonopsis pilosula* (Franch.) Nannf. (30 g), *Schisandra chinensis* (Turcz.) Baill. (6 g), *Ophiopogon japonicus* (Thunb.) Ker Gawl. (10 g), *Anemarrhena asphodeloides* Bunge (10 g), *Rehmannia glutinosa* (Gaertn.) DC. (10 g), *Reynoutria multiflora* (Thunb.) Moldenke (10 g), *Leonurus japonicus* Houtt. (10 g), *Angelica sinensis* (Oliv.) Diels (10 g), *Epimedium brevicornu* Maxim. (10 g), *Cinnamomum verum* J.Presl (3 g), *Scolopendra subspinipes mutilans* (2 g)*, Ephedra sinica* Stapf (3 g). Prepared by Cao et al., 2011	Randomized experiment	42	--	YQYYHXD(1 prescription/day)+Hypoglycemia agent	--	3 months	Prepared according to ChinaPharmacopoeia	Cao et al. (2011)
Fufang Xuanju capsule	Capsule	*Formica fusca* Linnaeus, *Epimedium brevicornu* Maxim	Randomized controlled experiment	30	30	Fufang Xuanju capsule (3 times/day, 3 capsules for each time) + Hypoglycemia agent	Liuwei Dihuang Pill (3 times/day, 3 pills for each time + Hypoglycemia agent	3 months	Prepared according to ChinaPharmacopoeia	You et al. (2017)
		*Cnidium monnieri* (L.) Cusson, *Lycium barbarum* L								
		Purchased from Zhejiang Strong Pharmaceutical Co., Ltd. (0.4 g/one capsule)								
			Randomized controlled experiment	40	40	Fufang Xuanju capsule (3 times/day, 3 capsules for each time) +Tadalafil (10 mg)	Tadalafil (10 mg/day)	4 weeks	Prepared according to China Pharmacopoeia	Jiang et al. (2016)
Ziyin Zhuangyang capluse	Capsule	*Rehmannia glutinosa (Gaertn.)* DC.(20 g), *Reynoutria multiflora* (Thunb.) Moldenke (18 g), *Lycium barbarum* L. (18 g), *Dioscorea oppositifolia* L. (15 g), *Broussonetia papyrifera* (L.) L’Hér. Ex Vent. (15 g), *Dendrobium nobile* Lindl.(15 g), *Ligustrum lucidum* W.T.Aiton (15 g), *Epimedium brevicornu* Maxim. (10 g), *Cuscuta chinensis* Lam. (10 g), *Cervus nippon* Temminck (10 g)*, Rosa laevigata* Michx. (10 g), *Albizia julibrissin* Durazz (8 g). Prepared by Yang and Dai, 2017	Randomized controlled experiment	30	30	Ziyin Zhuangyang capluse (1 prescription/day) +Hypoglycemia agent	Liuwei Dihuang Decoction+ Hypoglycemia agent	2 weeks	Prepared according to China Pharmacopoeia	Wu et al. (2015)
Qianlieshutong capsules	Capsule	*Phellodendron chinense* C.K.Schneid., *Paeonia lactiflora* Pall., *Angelica sinensis* (Oliv.) Diels, *Conioselinum anthriscoides* ‘Chuanxiong’, *Smilax glabra* Roxb	Randomized controlled experiment	27	27	Qianlieshutong capsules (3 times/day, 3 capsules for each time) + insulin injection	Thioctic acid injection (20 ml, 0.6 g/day) + insulin injection	3 weeks	Prepared according to China Pharmacopoeia	Guo, (2016)
		*Sparganium stoloniferum* (Buch.-Ham. Ex Graebn.) Buch.-Ham. Ex Juz., *Alisma plantago-aquatica subsp. Orientale* (Sam.) Sam., *Portulaca oleracea* L., *Verbena officinalis* L., *Saxifraga stolonifera* Curtis, *Bupleurum falcatum* L., *Cyathula officinalis* K.C.Kuan, *Glycyrrhiza uralensis* Fisch. Ex DC. Purchased from Baoding Tiaohao Pharmaceutical Co., Ltd. (0.4 g/one capsule)	Randomized controlled experiment	41	41	Qianlieshutong capsules (3 times/day, 3 capsules for each time) + insulin injection	Thioctic acid injection (20 ml, 0.6 g/day) + insulin injection	3 weeks	Prepared according to China Pharmacopoeia	He and Hao, (2018)
Yougui pill	Pill	*Rehmannia glutinosa* (Gaertn.) DC., *Cornus officinalis* Siebold & Zucc., *Dioscorea nipponica* Makino, *Lycium chinense* Mill., *Cervus nippon* Temminck, *Cuscuta chinensis* Lam., *Eucommia ulmoides* Oliv., *Angelica sinensis* (Oliv.) Diels, *Cinnamomum verum* J.Presl, *Aconitum carmichaeli* Debeaux. Purchased from Jiangxi Yintao Pharmaceutical Co., Ltd. (0.45 g/one capsule)	Randomized controlled Experimen	14	14	Yougui pill (3 times/day, 4 capsules for each time) + Hypoglycemia agent	Tadalafil (10 mg/day) + Hypoglycemia agent	8 weeks	Prepared according to China Pharmacopoeia	Zhia et al. (2020)
			Randomized controlled experimen	43	43	Yougui pill (3 times/day, 4 capsules for each time) + Hypoglycemia agent	Testosterone undecanoate soft Capsules (3 times/day, 1 capsules for each time) + Hypoglycemia agent	8 weeks	Prepared according to China Pharmacopoeia	Wang, (2018)

Huoxue Tongluo Qiwei Decoction (HXTLQWD) is composed of 12 types of Chinese medicines (Table 1). In a clinical trial, it showed that the IIEF-5 in the patients treated with HXTLQWD combined with tadalafil was significantly higher than that in the control group treated only with tadalafil, and total efficacy rate was higher in the treatment group than in the control group (81.8 *vs*. 51.5%) (Sun et al., 2021).

Yishen Huoxue Decoction (YSHXD) is composed of 16 Chinese medicines (Table 1) and is widely used to improve the semen quality of elderly men (Li et al., 2021). In a clinical trial, it showed that the IIEF-5 score and Erection Quality Scale (EQS) were higher in the group treated with YSHXD combined with a hypoglycemic drug than in the control group treated only with the hypoglycemic drug (Li et al., 2011). Another randomized control trial showed that YSHXD can significantly improve the erectile function of DM patients via reducing the serum level of endothelin-1(ET-1) and increasing that of NO, which is associated with suppression of oxidative stress (Zhang and Qiu, 2017).

Yiqi Yangyin Huoxue Decoction (YQYYHXD) is composed of 13 Chinese medicines (Table 1). A clinical study showed that YQYYHXD improves erectile dysfunction in type II DM patients by increasing the serum level of testosterone and reducing the levels of luteinizing hormone (LH) and estradiol (E2) (Cao et al., 2011).

Fufang Xuanju capsule is another example of TCM remedy and is composed of 4 Chinese medicines (Table 1). A randomized clinical trial enrolled 80 patients, who were then divided into control and treatment groups. After 4 weeks of treatment, the IIEF-5 score of the patients in the group treated with Fufang Xuanju capsule combined with tadalafil was significantly higher than that in the control group treated only with tadalafil (Jiang et al., 2016). Furthermore, another clinical study showed that Xuanju capsule can improve erectile function of DM patients *via* reducing serum levels of AGEs and Ang II (You et al., 2017).

Ziyin Zhuangyang capsule is extracted from 12 Chinese medicines (Table 1). In a clinical study involving 60 DM patients, after 8 weeks of treatment with Ziyin Zhuangyang capsule, the IIEF-5 score of the patients substantially increased, and the erectile function was also improved, compared with the levels before the treatment (Wu et al., 2015). Another study demonstrated that Ziyin Zhuangyang capsule and sildenafil have obvious synergistic effects in alleviating DED and can improve the sexual self-confidence and life quality of patients (Yang and Dai, 2017). However, the underlying mechanisms were not investigated in these studies.

Qianlieshutong capsule is extracted from 12 Chinese herbs (Table 1). In a randomized control clinical trial, after 3 months of treatment with Qianlieshutong capsule combined with *α*-Lipoic acid, it could significantly improve erectile function in DM patients via upregulating NO, superoxide dismutase (SOD), and glutathione peroxidase (GSH) and downregulating homocysteine, MDA, and ET-1, compared with the patients treated only with *α*-Lipoic acid (Guo, 2016). Another clinical study involving 82 patients in a course of 3 weeks of treatment showed that Qianlieshutong capsule can significantly improve the IIEF-5 score and increase the serum levels of SOD, GSH, NOS, and NO in DED patients (He and Hao, 2018).

Yougui pill is a 10-component TCM remedy (Table 1) and is well known in TCM for its therapeutic effects on impotence. With the development of research, it also was used to effectively treat sexual dysfunction (Wang Y. et al., 2022). A randomized clinical trial involving 42 patients of DED found that 6 weeks of treatment with Yougui pill could significantly increase the IIEF-5 score of the patients compared with those of the placebo-treated group (Zhia et al., 2020). Although the IIEF-5 score of the patients treated with Yougui pill was lower than the patients orally treated with tadanafil, adverse reactions were lower in the Yougui pill group than in the tadanafil group. Additionally, Yougui pill enhances erectile function via upregulating testosterone (Wang, 2018).

*Tribulus terrestris* L. (TT) is a plant of the Zygophyllaceae family, and has been used for a long time in China. In a prospective, randomized, double-blind, placebo-controlled clinical trial, the IIEF-5 score was significantly better in the TT-treated ED patients than in the placebo-treated patients. Additionally, significant improvement in sexual function was observed in the TT-treated ED patients versus the placebo group (Kamenov et al., 2017). However, for DED patients, another prospective, randomized, double-blind trial showed that TT is not more effective than placebo in improving the symptoms of ED or total serum testosterone level (Santos et al., 2014). Therefore, further investigation is needed to elucidate the underlying mechanism of the protective effect of TT on DED.

### The mechanisms whereby TCM ameliorates DED

Since TCM contain various chemical components and have numerous nutrition-related and pharmacological effects, the action mechanisms, pharmacokinetics, and therapeutic targets of most Chinese medicines are still elusive. There is currently no randomized controlled trial with a large sample size performed to evaluate the therapeutic effect of a TCM remedy on DED. However, numerous experimental studies, including animal studies and *in vitro* studies, have investigated the pharmacological mechanisms of various Chinese medicines in treating DED (Table 2). As discussed above, these biological pathways are interconnected, and these natural products may function in a multi-component and multi-target manner.

**TABLE 2:** The mechanisms by which Traditional Chinese medicine treat DED.

Property	Natural products	Type of study	Treatment method and sample size			Treatment	Targets or pathways	Chemical analysis	References
			Experiment group	Negative control group	Positive control group	Duration			
Rescued endothelial function	Huoxue Tongluo Qiwei Decoction (HXTLQWD)	Diabetes rat induced by STZ (55 mg/kg, i.p.)	HXTLQWD (7.8125 or 31.25 g/kg/day, suspension with deionized water) by gavage (n = 20)	The same volume of deionized water by gavage (n = 10)	LY333531 inhibitor (10 mg/kg) by gavage (n = 10)	8 weeks	Inhibit PKC signaling pathway	HPLC-MS	Li et al. (2022)
	Purchased from Beijing Kangrentang Pharmaceutical Co., Ltd								
	Tianjing Tongluo Decoction (TJTLD)	Diabetes rat induced by STZ (50 mg/kg, i.p.)	TJTLD (36 g/kg/day) by gavage (n = 10)	The same volume of saline by gavage (n = 10)	Tadalafil (0.2 mg/kg/time, two time/week) by gavage (n = 10)	4 weeks	Increase the release of sexual hormone levels	--	Shang et al. (2019a)
	Purchased from Beijing Tongrentang Pharmaceutical Co., Ltd	Diabetes rat induced by STZ (50 mg/kg, i.p.)	TJTLD (9.3 g/kg/day) by gavage (n = 10)	The same volume of saline by gavage (n = 10)	Tadalafil (0.2 mg/kg/time, two time/week) by gavage (n = 10)	4 weeks	Increase the activity of eNOS enzyme	--	Shang et al., 2019b
		Diabetes rat induced by STZ (50 mg/kg, i.p.)	TJTLD (9.3 g/kg/day) by gavage (n = 10)	The same volume of deionized water by gavage (n = 10)	Tadalafil (0.2 mg/kg/day) by gavage (n = 10)	4 weeks	Inhibit the RhoA/Rho-kinase pathway	--	Yu et al. (2020)
	Gross saponins extracted form of Tribulus terrestris L.(GSTT)	Diabetes rat induced by STZ (50 mg/kg, i.p.)	GSTT (40 mg/kg/d) by gavage (n = 6)	The same volume of purified water	Sildenafil (5 mg/kg/day) by gavage (n = 6)	4 weeks	Increase eNOS activity	--	Zhang et al. (2019a)
	Purchased from Xi’an Wanfang Biotech Co.,			by gavage (n = 6)					
	Ltd.								
	Extract of Eucommia ulmoides Oliv. Leaf (EULE)	Diabetes rat induced by STZ (30 mg/kg, i.p.)	EULE (0.5%, 1%, 2% w/w mixed into high-fat diet) (n = 30)	The same volume of purified water	Sildenafil (3 mg/kg/day) by gavage (n = 10)	16 weeks	Increase the level of nitric oxide (NO) and activate Akt-eNOS pathway	UPLC	Fu et al. (2019)
	Prepared by Fu et al., 2019			by gavage (n = 10)					
	Yiyuanqiwei Pills	Diabetes rat induced by STZ (40 mg/kg, i.p.)	Yiyuanqiwei Pills (1.5, 3, 6 g/kg/day) by gavage (n = 30)	The same volume of saline by gavage (n = 10)	Sildenafil (5 mg/kg/day) by gavage (n = 10)	2 months	Increase nNOS activity, activate NO- cGMP signaling	--	Wang et al. (2022a)
	Prepared by Wang et al., 2022a								
	Wuzi Yanzong recipe	Diabetes rat induced by STZ (40 mg/kg, i.p.)	Wuzi Yanzong recipe (1.08 g/kg/day) by gavage (n = 10)	The same volume of purified water	--	30 days	Activate NO-cGMP signaling	--	Cao et al. (2018a)
	Prepared by Cao et al., 2018a			by gavage (n = 10)					
Anti-atherosclerosis	*Achyranthes bidentata* Blume (ABR, 15 g) and *Gypsophila vaccaria* (L.)			The same volume of purified water		8 weeks	Reduce vascular endothelial growth factor A	HPLC	Wang et al. (2021a)
	Sm. (SV, 10 g)			by gavage (n = 6)	--				
	Purchased by Beijing Kangrentang Pharmaceutical Co., Ltd	Diabetes rat induced by STZ (55 mg/kg, i.p.)	ABR-SV suspension (2.5 g/kg/day) by gavage						
Anti-oxidative properties	Panax notoginseng saponins (PNS) Purchased from Weikeqi Biological Technology	Diabetes rat induced by STZ (60 mg/kg, i.p.)	PNS (50, 100 and 150 mg/kg/day) by i.p. (n = 18)	The same solutions of saline daily by i.p. (n = 6)	--	4 weeks	Increase SOD activity	--	Li et al. (2014)
	Danshen injection	Diabetes rat induced by STZ (60 mg/kg, i.p.)	Danshen injection (0.5 or 1 ml/kg/day) by i.p. (n = 20)	The same solutions of saline daily by i.p. (n = 10)	--	6 weeks	Increase SOD activity and reduce ROS	HPLC	Zhang et al. (2018)
	Purchased from Chiatai Qingchunbao Pharmaceutical Co., Ltd								
	Hirudo nipponica Whitman. (14 g) and Scolopendra subspinipes mutilans L. Koch (7 g)	Diabetes rat induced by STZ (55 mg/kg, i.p.)	The leech-centipede” suspension (0.15, 0.3, 0.6 g/kg/day) by gavage (n = 18)	The same volume of purified water	Tadalafil (0.5 mg/kg/day) by gavage (n = 6)	8 weeks	Increase SOD activity	UPLC-MS	Wang et al. (2021b)
	Purchased by Beijing Kangrentang Pharmaceutical Co., Ltd			by gavage (n = 6)					
	Yougui Capsule	Diabetes rat induced by STZ (60 mg/kg, i.p.)	Yougui Capsule (1.2, 2.4, 3.6 g/kg/day) by gavage (n = 39)	The same volume of purified water	--	12 weeks	Increase the activity of SOD and GSH	--	Liu et al. (2012)
	Purchased from Jiangxi Yintao Pharmaceutical Co., Ltd. (0.45 g/one capsule)			by gavage (n = 11)					
Anti-apoptosis	Bushen HuoXue Decoction (BSHXD)	Diabetes rat induced by STZ (55 mg/kg, i.p.)	BSHXD (3, 6 g/kg/day) by gavage (n = 13)	The same volume of saline by gavage (n = 8)	--	8 weeks	Activate protein kinase B	--	Yue et al. (2015)
	Prepared by Yue et al., 2015	Diabetes rat induced by STZ (40 mg/kg, i.p.)	Wuzi Yanzong recipe (1.08 g/kg/day) by gavage (n = 10)	The same volume of purified water		30 days	Reduce the expression of cleaved caspase-3	--	Cao et al. (2018a)
	Wuzi Yanzong recipe			by gavage (n = 10)					
	Prepared by Cao et al., 2018a								
	Danshen injection	Diabetes rat induced by STZ (60 mg/kg, i.p.)	Danshen injection (0.5 or 1 ml/kg/day) by i.p. (n = 20)	The same solutions of saline daily by i.p. (n = 10)		6 weeks	Reduce the expression of cleaved caspase-3 and GADD153	HPLC	Zhang et al. (2018)
	Purchased from Chiatai Qingchunbao Pharmaceutical Co., Ltd								
	Ganoderma lucidum polysaccharide	Diabetes rat induced by STZ (60 mg/kg, i.p.)	Ganoderma lucidum polysaccharide (100, 400 mg/kg/day) by gavage (n = 6)	The same volume of purified water	Metformin (20 mg/kg/day) (n = 3)	8 weeks	Reduce the expression of cleaved caspase-3	--	Yao and Zhu, (2022)
	Purchased from Shanghai yuanye Bio-TechnologyCo., Ltd.			by gavage (n = 3)					
	Panax notoginseng saponins (PNS)	Diabetes rat induced by STZ (60 mg/kg, i.p.)	PNS (50, 100 and 150 mg/kg/day) by i.p. (n = 18)	The same solutions of saline daily by i.p. (n = 6)	--	4 weeks	Increase the expression of Bcl-2	--	Li and Gou, (2013)
	Purchased from Weikeqi Biological Technology								

eNOS: endothelial nitric oxide synthase; GADD153: growth arrest and DNA, damage-inducible gene 153; high-performance liquid chromatography-mass spectrometry: HPLC-MS; NOS: neuronal nitric oxide synthase; PKC: protein kinase C; ROS: reactive oxygen species; SOD: superoxide dismutase; STZ: streptozocin; UPLC: ultra performance liquid chromatography; UPLC-MS: Ultra Performance Liquid Chromatography-mass spectrometry.

### Rescued endothelial function

As discussed above, HXTLQWD can stimulate blood circulation and dredge collaterals, remove blood stasis, and calm wind. A recent study revealed that HXTLQWD can downregulate the protein and mRNA levels of PKC, rescue the function of endothelial cells and ultrastructure of the corpus cavernosum, and improve the erectile function of rats with DED (Li et al., 2022).

Tianjing Tongluo Decoction (TJTLD) is a Chinese herbal remedy composed of 4 Chinese medicines [*Epimedium brevicornu* Maxim.(15 g)*, Rehmannia glutinosa* (Gaertn.) DC. (10 g)*, Hirudo nipponica* Whitman (10 g). and *Cyathula officinalis* K.C.Kuan (10 g) Prepared according to China pharmacopoeia]. A previous study showed that TJTLD has significantly beneficial effects on improving erectile function in diabetic rats and not only significantly promotes the sexual function via increasing the licking and sniffing times, the first back-climbing time, the straddling time, and insertion frequency but also upregulates serum testosterone level (Shang et al., 2019a). Although the underlying molecular mechanisms were not investigated in this study, two other studies showed that TJTLD could increase the activity of eNOS in penile spongiform and inhibit the RhoA/Rho-kinase pathway to rescue the endothelial function (Shang et al., 2019b; Yu et al., 2020).

Gross saponins extracted from *Tribulus terrestris* L. (GSTT) are the main active ingredients of *Tribulus terrestris* L. (TT), which is widely used to treat impotence. Previous studies have shown that TT has multiple biological effects, including improving glycolipid metabolism, attenuating oxidative stress, and inhibiting apoptosis (Zhang et al., 2010; Ercan and El, 2016). According to the study by Zhang et al., GSTT improves penile endothelial function via upregulating eNOS (Zhang H. et al., 2019).

*Eucommia ulmoides* Oliv. is used as a traditional medicine in China. The water extract of *Eucommia ulmoides* Oliv. leaf, generally known as Du-zhong tea, is a popular beverage in Asia and has also been used as a new edible remedy against hypertension (Zhu and Sun, 2018). A previous study showed that administration of *Eucommia ulmoides* Oliv. leaf extract significantly upregulates nitric oxide (NO) and cyclic guanosine monophosphate (cGMP) and activates the Akt-eNOS pathway to restore endothelial function of rat model of DED (Fu et al., 2019).

Yiyuanqiwei Pills is a classical herbal formula that stimulates blood circulation and nourishes blood. It is composed of approximately 20 Chinese medicines, namely *Rehmannia glutinosa* (Gaertn.) DC. (25 g), *Cistanche deserticola* Ma (20 g)*, Cervus nippon* Temminck (20 g), *Eucommia ulmoides* Oliv. (15 g), *Cuscuta chinensis* Lam (15 g), *Cullen corylifolium* (L.). Medik. (15 g), *Cynomorium coccineum subsp. songaricum* (Rupr.) J.Léonard (15 g), *Lycium barbarum* L. (15 g), *Epimedium brevicornu* Maxim.(15 g), *Panax ginseng* C.A.Mey.(15 g), *Achyranthes bidentata* Blume.(15 g), *Asparagus cochinchinensis* (Lour.) Merr. (15 g), *Hippocampus trimaculatus* Leach. (1 pair), *Bombyx mori* Linnaeus. (10 g), *Anax parthenope* Selys. (9 g), *Aspongopus chinensis* Dallas. (9 g), *Succinum* (8 g), *Syzygium aromaticum* (L.) Merr. & L.M.Perry (8 g), *Wurfbainia villosa* var. villosa (8 g), and *Dolomiaea costus* (Falc.) Kasana & A.K.Pandey (9 g). According to Wang *et al*, Yiyuanqiwei pills can improve penile erection function in diabetic rats and reduce the pathological damage in the penile cavernous body (Wang D. et al., 2022). Especially, transmission electron microscopy observations have revealed that in diabetic rats, the blood sinus endothelium is broken, the blood sinus cavity is narrowed, and the endothelial cells are swollen, but these pathological features are significantly ameliorated by Yiyuanqiwei Pills treatment, in association with upregulation of nNOS and eNOS and activation of the NO-cGMP signaling pathway (Wang D. et al., 2022).

Wuzi Yanzong recipe is a classical TCM prescription and is composed of 5 types of Chinese medicines [*Cuscuta chinensis* Lam. (40 g), *Lycium dasystemum* Pojark. (40 g), *Rubus idaeus* L. (20 g), *Plantago ovata* Forssk. (10 g) and *Schisandra chinensis* (Turcz.) Baill. (5 g). Prepared according to China pharmacopoeia]. It was reported that 4 months treatment of Wuzi Yanzong recipe could improve sperm quality via activating NO-cGMP pathway (Cao et al., 2018a).

### Anti-atherosclerotic properties

*Achyranthes bidentata* Blume and *Gypsophila vaccaria* (L.) Sm. are two common Chinese herbs that are widely used in the clinical treatment of DM patients via strengthening the renal function and promoting blood circulation (Ma et al., 2017). It has been shown that vascular endothelial growth factor A (VEGFA) and angiotensin-converting enzyme (ACE) are upregulated in the corpus cavernosum of diabetic rats, and these proteins can promote the generation of mucosal blood vessels and increase the risk of vascular thrombosis (Scaldaferri et al., 2009). However, *Achyranthes bidentata* Blume and *Gypsophila vaccaria* (L.) Sm. can downregulate these two proteins (Wang et al., 2021a). Additionally, in comparison with healthy rats, the number of blood-containing sinuses in the cavernous body of diabetic rats is significantly reduced, accompanied by a reduction in the densities of endothelial and smooth-muscle cells. Combinatorial treatment with *Achyranthes bidentata Blume and Gypsophila vaccaria (L.) Sm.* increases the density of endothelial cells (Wang et al., 2021a).

### Anti-oxidative properties

Panax notoginseng saponins (PNS), extracted from a Chinese herb named *Panax notoginseng* (Burkill) F.H.Chen, has an antioxidant activity. It has been reported that PNS improves erectile function in diabetic rats via increasing the activity of SOD and rescuing the functions of the endothelial and smooth-muscle cells in the penis (Li et al., 2014).

Danshen, a traditional Chinese herbal medicine, is the dried root of the plant *Salvia miltiorrhiza* Bunge. Accumulating studies have demonstrated that injection of Danshen has antioxidative effects in animal experiment (Raisi et al., 2021; Yuen et al., 2021). Further investigation has revealed that Danshen injection increases the activity of superoxide dismutase (SOD) and reduces the levels of reactive oxygen species (ROS) in the penile tissue of diabetic rats, thereby improving the erectile dysfunction (Zhang et al., 2018).

*Hirudo nipponica* Whitman. (leech) and *Scolopendra subspinipes mutilans* L. Koch (centipede) are used as two common TCM therapeutics. In a study, leech- or centipede-derived therapeutics could improve ED by inhibiting the expression of PKC upon 8 weeks of treatment, and this beneficial effect was found associated with attenuating oxidative-stress injury *via* increasing SOD activity (Wang et al., 2021b). Additionally, Yougui capsule has been shown to improve ED by enhancing the activity of SOD and GSH in the cavernous tissue of diabetic rats (Liu et al., 2012).

### Anti-apoptotic properties

Bushen HuoXue Decoction (BSHXD) is a Chinese herbal compound composed of 5 Chinese medicines [*Cistanche deserticola* Ma (10 g), *Cervus nippon* Temminck (2 g), *Carthamus tinctorius* L. (9 g), *Panax ginseng* C.A.Mey. (9 g), and *Cyathula officinalis* K.C.Kuan (6 g), Prepared according to China pharmacopoeia]. In a previous study, administration of BSHXD to a rat model of DM for 4 weeks could inhibit the apoptosis of the cells in the corpus cavernosum smooth muscle via activating protein kinase B (AKT) (Yue et al., 2015).

It was reported that Wuzi Yanzong recipe could improve the sperm quality and rescue the testicular dysfunction in old rats via decreasing germ-cell apoptosis (Zhao et al., 2019). An animal study showed that Wuzi Yanzong recipe can substantially improve the reproductive and erectile function in DED, possibly by reducing the apoptosis of spermatogenic cells (Cao et al., 2018a). Another study revealed that Wuzi Yanzong recipe can reduce the blood sugar level and improve the pathological changes in the testicular tissue (Cao et al., 2018b). Furthermore, Wuzi Yanzong recipe exerts an anti-apoptotic effect via attenuating oxidative damage (Ma et al., 2018).

Furthermore, Danshen injection also reduces the number of apoptotic cells in the corpora cavernosum via suppressing caspase-3 activation and the expression of “growth arrest and DNA damage-inducible gene 153” (GADD153) (Zhang et al., 2018).

Ganoderma lucidum polysaccharides are the main bioactive ingredients of *Ganoderma lucidum* (Curtis) P. Karst. It has been reported that Ganoderma lucidum polysaccharides can inhibit the apoptosis of sponge smooth muscle cells by suppressing caspase-3 activation and thereby improves erectile function in diabetic rats (Yao and Zhu, 2022). Furthermore, PNS protects endothelial and smooth-muscle cells from apoptosis via upregulating Bcl-2 and protects the function of penile cells, thereby improving erectile function in diabetic rats (Li and Gou, 2013).

## Conclusions and perspectives

As mentioned above, the pathogenesis of DED is more complex than ED, and the development of DED involves multiple risk factors. Therefore, multi-target treatments may be more effective in treating DED than targeting a single factor. However, recent clinical studies have indicated that chemical drugs do not have the ideal therapeutic effects. Therefore, the application of TCM to DED treatment is becoming a new trend. According to previous studies, Chinese herbs have multiple pharmacological activities and physiological effects, including anti-oxidative, anti-apoptotic, and anti-atherosclerotic effects and rescue endothelial function. However, there are also several limitations in the published studies.

First, the sample size is too small in most of the clinical studies. There is a lack of large scale, multicenter, randomized, and controlled clinical trials on the use of these drugs to treat DED. Second, some of the findings still await verification through clinical studies, and although the clinical trials conducted to date have confirmed that the clinical symptoms are somewhat improved, the underlying mechanisms are still ambiguous. Third, there are also some toxic effects of Chinese herbs. According to the Chinese pharmacopoeia, some TCM are indeed toxic which could cause damage to the renal, nervous, respiratory, and reproductive system (Cai et al., 2019). However, published *in vivo* studies seldom mention the adverse effects of the treatments. In order to reduce the toxicity of TCM, the dosage of TCMs should not exceed the limit prescribed by the current China Pharmacopoeia. Fourth, the long effect of TCM on DED is not investigated in the current clinical studies, since TCM cannot be taken continuously. Fifth, TCM is often criticized and rejected by Western scientists, since the mechanisms of action, complex pharmacokinetics targets have not been fully classified and most of TCM formulae prescribed by doctors contain varied amounts of components for different person. Therefore, the clinical trials were mainly studied in China or east Asian countries. Finally, DED is caused by multiple factors; however, studies have mostly focused on examining the effects of TCM on one or a few aspects of DED.

In order to overcome these limitations, we believe that future research interests should be focused on at least two aspects. One is integrating TCM with network pharmacology analysis. It is well known that network pharmacology is a new subject which could analysis to search the key compounds and targets, and experimental verification to ensure the reliability of the predicted results (Luo et al., 2020). As discussed above, due to the complexity of TCM components and the interactions between TCM components and disease targets, it is difficult to fully classify the underlying molecular mechanism of TCM, that is also the major reason why western countries reject TCM. Indeed, some progress has been made in the TCM network pharmacology in recent years, including predicting herb targets, understanding biological foundation of diseases and syndromes, network regulation mechanisms of herbal formulae (Wang X. et al., 2021). However, there is few reports to investigate the role of TCM network pharmacology on DED. Second, there was no standardize the clinical research process to perform a large scale, multicenter and controlled trials. Therefore, it urgently needs to establish a unified standard for clinical research on DED.
